# Prediction accuracy for feed intake and body weight gain using host genomic and rumen metagenomic data in beef cattle

**DOI:** 10.1186/s12711-025-01007-8

**Published:** 2025-10-30

**Authors:** Andrew Lakamp, Seidu Adams, Larry Kuehn, Warren Snelling, James Wells, Kristin Hales, Bryan Neville, Samodha Fernando, Matthew L. Spangler

**Affiliations:** 1 Department of Animal Sciences, University of Nebraska-Lincoln, Lincoln, NE 68583 USA; 2 USDA-ARS, U.S. Meat Animal Research Center, Clay Center, NE 68933 USA

## Abstract

**Background:**

Host genomic and rumen metagenome data can predict feed efficiency traits, supporting management decisions and increasing profitability. This study estimated the proportion of variation of average daily dry matter intake and average daily gain explained by the rumen metagenome in beef cattle, evaluated prediction accuracy using genomic data, metagenomic data, or their combination, and explored methods for modelling the rumen metagenome to improve phenotypic prediction accuracy. Data from 717 animals on four diets (two concentrate-based and two forage-based) were analyzed. Animal genotypes consisted of 749,922 imputed sequence variants, while metagenomic data comprised 16,583 open reading frames from ruminal microbiota. The metagenome was modelled using six (co)variance matrices, based on combinations of two creation methods and three modifications. Nineteen mixed linear models were used per trait: one with genomic effects only, six with metagenomic effects, six combining genomic and metagenomic effects, and six adding interaction effects. Two cross-validation schemes were applied to evaluate prediction accuracy: fourfold cross-validation balanced for diet type with 5 replicates and leave-one-diet-out cross-validation, where three diets served as training and the fourth as testing. Prediction accuracy was measured as the correlation between an animal’s summed random effects and its adjusted phenotype.

**Results:**

Although minimal, differences existed in parameter estimates and validation accuracy depending on how the metagenome effect was modelled. Median phenotype prediction accuracy ranged from −0.01 to 0.28. No specific set of model characteristics consistently lead to the highest accuracies. Models which combined genome and metagenome data outperformed those using either data source alone. Models where the rumen metagenome (co)variances matrix was scaled within each diet composition generally led to lower prediction accuracies in this study.

**Conclusions:**

The rumen metagenome can explain a significant proportion of variation in beef cattle feed efficiency traits. Those traits can also be predicted using either host genome or rumen metagenome, though using both sources of information proved more accurate. Multiple methods of forming the metagenome (co)variance matrix can lead to similar prediction accuracies.

**Supplementary Information:**

The online version contains supplementary material available at 10.1186/s12711-025-01007-8.

## Background

In the beef cattle industry, feed efficiency and its constituent traits, average daily gain (ADG) and average daily dry matter intake (ADDMI) play a large role in the profitability of an operation [1]. Simulations have demonstrated that when ADG increases, profitability tends to increase across all sectors of the beef industry [2]. Therefore, to better manage a beef operation and make decisions relative to managing and marketing individual animals, estimates more accurate than phenotypic evaluation regarding the production performance of a given animal would be beneficial. It has been well-established that ADG and ADDMI can be manipulated through genetic selection [3–5]. Therefore, phenotypic predictions can be accomplished using genetic information.

Microorganisms are what allow ruminant animals to obtain energy and nutrients from what would otherwise be indigestible matter (i.e., lignocellulosic plant material). Through the process of fermentation, microbes deliver up to 70% of the energy metabolized by a ruminant animal [6]. It has been demonstrated that beef cattle differing in feed efficiency differ in rumen microbiome composition [7–9] and that features of the microbiome are associated with feed efficiency phenotypes [10, 11]. To this end, rumen microbiome information can be used to help explain variation in host phenotypes, where the fraction of variation attributed to the microbiome is often denoted as microbiability (*m*^*2*^) [12]. In beef cattle, estimates of *m*^*2*^ averaged 0.11 for weight gain from birth to weaning, weight gain from weaning to fattening, and weight gain from fattening to slaughter [13].

Utilizing metagenomics or a combination of host genetic and metagenomic information for phenotype prediction has recently been an area of interest. Efforts have been made in swine [14–16], dairy cattle [17–21], beef cattle [7, 22, 23] and sheep [24, 25]. There have also been multiple studies examining the increase in accuracy of estimated breeding values (EBV) in beef cattle by including microbial information [11, 26, 27]. Predictions using microbial data often transform the data to reflect relative abundance of the microbial features to create a microbiome-based (co)variance matrix, or a metagenome relationship matrix (MRM), in the same way as demonstrated by Ross et al. [17]. Variations of creating the MRM have been documented [20]; however, those authors focused on the data used in the MRM (e.g., relative abundance vs. principal components), rather than the scaling of the (co)variance matrix itself. Consequently, knowledge of differing construction methods of the MRM, and the consequences on parameter estimation and predictions, is currently lacking.

The objective of this study was to address gaps in the literature involving the proportion of variation explained by the genetics of the rumen microbial community in feed efficiency related traits in beef cattle, assess the prediction accuracy of such traits using genomic data, metagenomic data, or a combination of the two, and explore methods of formulating the MRM in an attempt to increase phenotypic prediction accuracy.

## Methods

### Animals

Activities involving animals were approved by the Institutional Animal Care and Use Committee (IACUC) at the U. S. Meat Animal Research Center (USMARC). Over the course of 3 years at the USMARC Bovine Feed Efficiency Facility, 767 animals consisting of an admixture of 18 breeds from the Germplasm Evaluation (GPE) program were on test [28]. These animals were divided into steer and heifer groups. The steer groups received one of two high concentrate diets, while the heifer groups received one of two high forage diets. Steer diets 1 and 2 (SD1 and SD2) were comprised of alfalfa hay (8%), balance pellet rumensin (4.25%; Feedlot 40–20 (Kent Nutrition Group, Muscatine, IA, USA) medicated with 500 g/ton monensin), dry-rolled corn (57.35% and 77% for SD1 and SD2, respectively), urea (0.40% and 0.75% for SD1 and SD2, respectively), and wet distillers’ grain with solubles (WDGS; 30% and 10% for SD1 and SD2, respectively). Heifer diet 1 (HD1) was composed of 32% alfalfa hay, 30% alfalfa haylage, 25% dry-rolled corn, 3% soybean meal, and 10% WDGS. Whereas heifer diet 2 (HD2) was 35% each of alfalfa hay and alfalfa haylage with 8% dry-rolled corn, and 22% WDGS. Tests were targeted to be 63 days and all tests achieved at least 58 days in length. All animals were fed ad libitum and feed was delivered once daily in the morning. Intake was measured using an Insentec feeding system (Hokofarm Group, Emmeloord, the Netherlands). Average daily dry matter intake was the sum of the dry matter of each animal consumed divided by the number of days on test. Unshrunk body weight measurements were taken every 3 weeks with additional weights taken the first two and last two test days. Average daily gain was calculated by regressing body weight on the number of days on feed. Rumen samples were taken after a period of adaptation to the diet, at the end of the test, via esophageal tubing. The summary statistics for the phenotypes are in Table 1. Samples were collected in the morning before feeding. Samples were frozen in liquid nitrogen, transported to the laboratory, and stored in a −80 °C freezer until DNA extraction and sequencing. As multiple animals were sampled at the same time, the term sampling group denotes a collection of animals within the same year/season and sex designation. Sampling groups differ from management groups as sampling groups do not take implantation strategy or specific ration formulations into account.

**Table 1:** Summary statistics of average daily dry matter intake (ADDMI) in kg and average daily gain (ADG) in kg

Trait	No. Records	Minimum	Mean	Maximum	St. Dev
ADDMI	717	4.39	9.47	16.64	1.64
ADG	717	0.33	0.96	1.18	0.32

**Fig. 1:**
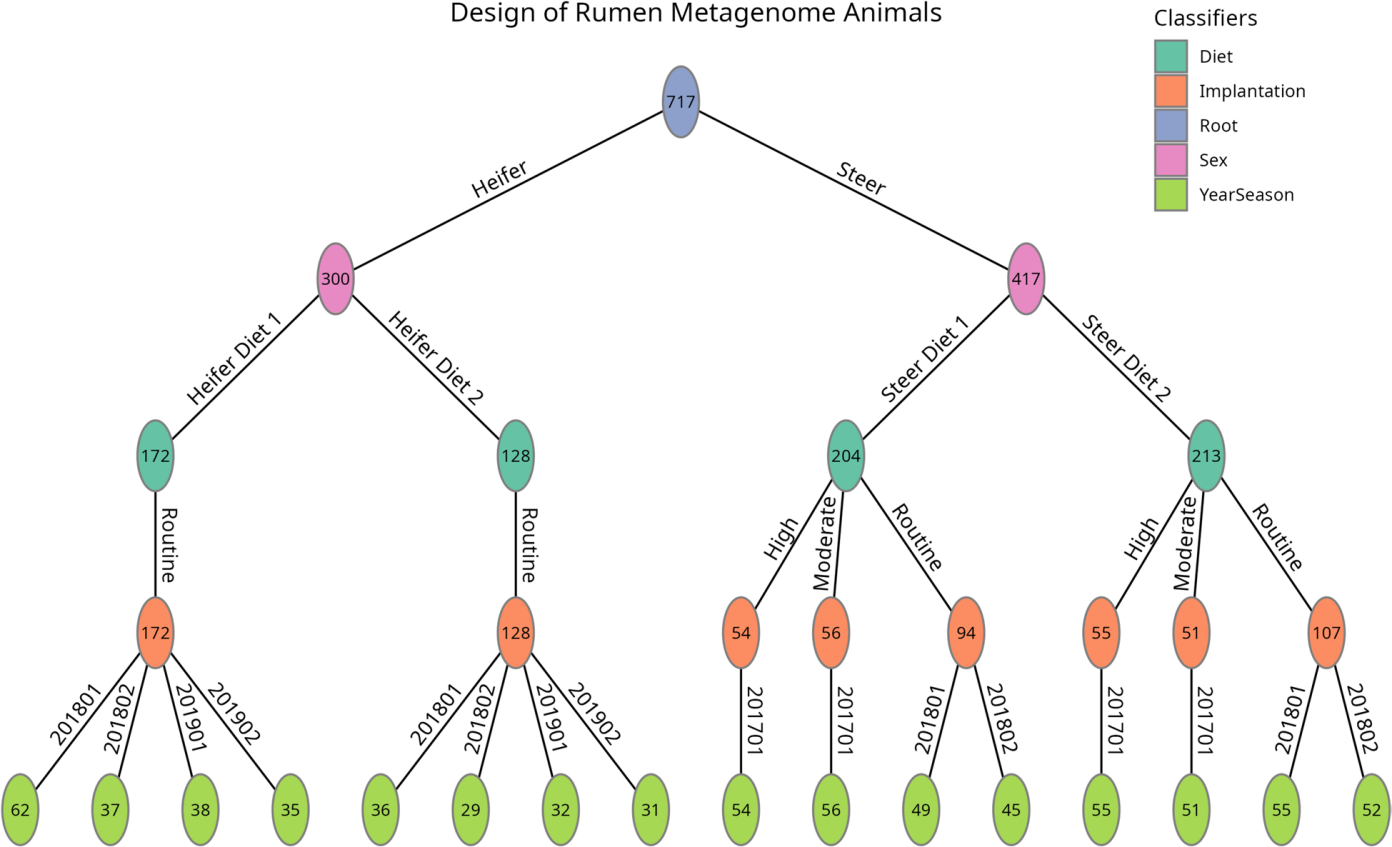
Graphical representation of the study design. Each effect is colored coded. Edges are labeled with details of the level of the fixed effect. Nodes contain the number of animals in that level. Management groups defined as a concatenation of sex, diet, implantation strategy, and year-season

Data for analysis were limited to those animals that had all genomic, metagenomic, phenotypic, and fixed effect information (breed fractions, management group, and expected heterozygosity estimates). Outliers, animals that had ADDMI or ADG further than three standard deviations away from the mean of either trait within a diet, were removed. In total, 717 animals remained for analysis. Data were hierarchical in design with animals being split into sex (diet type), then specific diet, growth implantation strategy, and finally year-season. This resulted in 16 management groups which were a concatenation of sex, diet, implantation strategy, and year-season (Fig. 1). Each management group represented between 29 and 62 different sires with a median of 47 sires. Breed fractions were defined as the proportion of each of 18 breeds, including breeds prominent in the United States and USMARC composites, in each animal.

### Host genomic data

Host genotypes were imputed from low-pass whole-genome sequences and commercial single nucleotide polymorphisms (SNP) array genotypes to a common set of approximately 1.14 million variants. First, low-pass sequence data were imputed to whole genome sequence with GLIMPSE [29] using a reference panel composed of 598 whole-genome sequences from the NCBI Sequence Read Archive and 348 from GPE bulls [30]. The snpEff software [31] was utilized to assess functionality using the Ensembl release 110 annotation [32] of the ARS-UCD1.2 assembly of the bovine genome [33]. Variants of interest (as determined by Snelling et al. [34]) in protein-coding sequences, untranslated regions of protein-coding genes, microRNA and other RNA sequences were identified and extracted. Single nucleotide polymorphisms probed by the Illumina BovineHD (Illumina, San Diego, CA, USA) and Neogen GGP-F250 (Neogen, Lincoln, NE, USA) were also extracted from the imputed genotype calls. All calls with greater than 75% confidence were coded as 0, 1, 2 copies of the alternate allele; calls below this threshold were coded as unknown. The threshold for genotype call confidence was determined based on a USMARC in-house comparison between the results of GLIMPSE imputation software [35] with a 75% confidence threshold (which was used herein) and the results from the loimpute method [29] with a 90% confidence threshold.

To get this same set of variants for animals without low-pass sequence, the genotypes of animals with one or more commercial SNP assays were extracted from the USMARC database. Of the 717 animals in the final dataset, 213 were genotyped on the GGP 50 K SNP array (Neogen, Lincoln, NE, USA) and 368 were genotyped on the GGP 100 K SNP array (Neogen, Lincoln, NE, USA). These array genotypes were re-coded to the number of alternate alleles based on the position and reference allele documentation curated by the National Animal Genome Research Program (NAGRP) [36]. Genotypes of animals across multiple platforms were collapsed into a single file with conflicting calls coded as unknown. The low-pass and array genotypes from all animals were combined into a single file. This file was added to pedigree data and input into findhap v3 [37]. The result was a common set of approximately 1.14 million SNP for both low-pass and array genotyped animals.

Lastly, the genotype calls for the subset of the 717 animals with complete data were subject to a filter of minor allele frequency of greater than 0.05. This left 749,922 variants available for downstream analysis.

### Microbial DNA extraction and sequencing

Extraction of the DNA was performed with the Mag-Bind Stool DNA 96 Kit (Omega Bio-tek, Inc., Norcross, GA) following the protocol in Paz et al. [38]. After extraction, DNA quality was checked using gel electrophoresis. Library preparation was performed using the NEBNext Ultra II DNA Library Prep Kit for Illumina (New England Biolabs, Ipswitch, MA) according to the manufacturer's instructions. Dual-barcoded adapters were applied, and the resulting libraries underwent polymerase chain reaction (PCR) cleanup and size selection targeted at 500 to 1200 base pairs using the Pippen Prep System with 1.5% gel cassettes (Sage Science, Inc.). Sequencing was conducted on the Illumina HiSeq platform (Illumina, San Diego, CA, USA). The 150 base pair paired-end strategy was used to achieve an average depth of ~ 20 million reads per sample.

### Bioinformatics

The Read_qc module of meta-WRAP [39] was used for quality control of the raw reads including removal of the host genome, human genome, and PhiX174 control sequences, along with adapter and length trimming. Afterwards, a total of 8.7 terabytes of clean data remained. Samples were pooled and co-assembled de novo using MEGAHIT [40] within sample groups. Prodigal v2.6 [41] was used to predict open reading frames (ORF), a stretch of DNA between a start and stop codon. A non-redundant gene catalog was made using CD-HIT v4.8 [42] based on 95% similarity, again within sampling group. The ORF catalogs from the groups were concatenated into a single file of approximately 43 million rumen ORF. Clean reads were mapped to the complete ORF catalog using BWA [43] and SAMtools [10] to generate an ORF abundance table. Any ORF shorter than 100 base pairs were removed as were any ORF with less than 10% prevalence and less than 0.01% abundance in at least one sample. This resulted in 16,583 ORF for analysis.

### Modeling of the random effects

Three types of random effects were considered: the host genomic effect, the metagenome effect, and the interaction of host genomic and metagenome effects.

The host genetic effect was modeled by constructing a genomic relationship matrix (GRM) using the first method of VanRaden [44]. Briefly, a matrix ($\mathbf{Q}$) of *n x s* (number of individuals by number of SNP) was constructed where the elements of the matrix are the number of alternate alleles each animal has for each locus. The GRM ($\mathbf{G}$) was formed using Eq. (1):

1 $${\mathbf{G}} = \frac{{\left( {{\mathbf{Q}} - {\mathbf{P}}} \right)\left( {{\mathbf{Q}} - {\mathbf{P}}} \right)^{\prime}}}{{2\mathop \sum \nolimits_{{{\text{i}} = 1}}^{{\text{s}}} {\text{p}}_{{\text{i}}} \left( {1 - {\text{p}}_{{\text{i}}} } \right)}},$$

where ${p}_{i}$ was the frequency of the alternate allele at locus $i$ such that $\mathbf{P}$ was a matrix with columns equal to ${2p}_{i}$. The division of $2{\sum }_{i=1}^{s}{p}_{i}(1-{p}_{i})$ scales the values of the cross-product by the sum of the SNP variances. For inversion, the GRM was tuned with a diagonal matrix with elements of 0.001 (${\mathbf{G}}_{\mathbf{T}}$ = $\mathbf{G}$ + 0.001$\mathbf{I}$).

One of the main objectives of the current study was to investigate the predictive power of different methods of modeling the metagenome effect. To that end, six MRM were created. Each MRM was constructed using combinations of a pair of characteristics, which were known as method (i.e., the formula used to construct the matrix) and adjustment (i.e., within management group scaling or weighting). Methods to construct the MRM were Method 1 (M1) derived from the first method of VanRaden [44] and Method 2 (M2) derived from Ross et al. [17]. In particular, these methods differ in how the MRM were scaled as detailed below. There were also adjustments (alterations) applied to these matrices that were intended to incorporate additional knowledge about the data. The naïve adjustment was the MRM with no additional modification (baseline). Given host genetics are known to influence the composition of the rumen microbiome (e.g., [7, 45]), the weighted adjustment was introduced in order to partially account for host genetic contribution to differences in relative abundance of ORF among animals by introducing a weighting factor of 1–h^2^ for each ORF. Lastly, the data-driven adjustment was included to account for the fact the animals in this dataset were fed differing diets. Diet, particularly forage concentration, is known to have a large effect on the composition of the rumen microbiome [46, 47].

All MRM began with a matrix of *n x m* (number of animals by number of ORF) denoted $\mathbf{S}$ where the elements of $\mathbf{S}$ were calculated from initial read counts using Eq. (2):

2 $${\mathbf{S}}_{{{\text{ij}}}} = {\text{log}}_{10} \left( {\frac{{{\mathbf{R}}_{{{\text{ij}}}} + 1}}{{\mathop \sum \nolimits_{{{\text{j}} = 1}}^{{\text{m}}} \left( {{\mathbf{R}}_{{{\text{ij}}}} + 1} \right)}}} \right),$$

where ${\mathbf{S}}_{\text{ij}}$ is the log-transformed relative abundance and ${\mathbf{R}}_{\text{ij}}$ is the read count of ORF $\text{j}$ for animal $\text{i}$, and $\text{m}$ is the number of ORF present across all samples.

The M1 MRM, ${\mathbf{M}}_{1}$, was constructed using Eq. (3):

3 $${\mathbf{M}}_{1} = \frac{{\left( {{\mathbf{S}} - {\mathbf{N}}} \right)\left( {{\mathbf{S}} - {\mathbf{N}}} \right)^{\prime}}}{{\mathop \sum \nolimits_{{{\text{j}} = 1}}^{{\text{m}}} {\text{var}}\left( {{\text{s}}_{{\text{j}}} } \right)}},$$

where $\mathbf{N}$ is an *m x n* matrix with columns being the arithmetic mean of ORF $j$ repeated n times, and the scaling factor is the sum of the variances for each ORF. This yielded an MRM that was directly analogous to a GRM constructed using the first method of VanRaden [44]. The modifications from the first VanRaden method were done to accommodate the distributional properties of the data in **S**. Log-transformed relative abundance data are approximately normally distributed as opposed to the binomial distribution of SNP data. Thus, for the construction of ${\mathbf{M}}_{1}$ a Gaussian mean and variance were assumed.

In ${\mathbf{M}}_{2}$, the columns of $\mathbf{S}$ were centered and scaled individually, creating a new matrix, $\mathbf{C}$.

4 $${\mathbf{C}}_{{{\text{ij}}}} = \frac{{{\mathbf{S}}_{{{\text{ij}}}} - \overline{{{\mathbf{S}}_{{.{\text{j}}}} }} }}{{\sqrt {{\text{var}}({\mathbf{S}}_{{\text{.j}}} )} }}.$$

The M2 MRM, or ${\mathbf{M}}_{2}$, described in Eq. (5):

5 $${\mathbf{M}}_{2} = \frac{1}{{\text{m}}}{\mathbf{CC^{\prime}}}.$$

where $m$ is the number of ORF. This construction method has often been employed in the literature for the creation of an MRM (e.g., [14]). Notably, this method is analogous to the second method of creating a GRM as shown by VanRaden [42].

The weighted adjustment was done by first creating a diagonal matrix ($\mathbf{D}$) with diagonal elements of 1–*h*^*2*^_ORF_, or 1 minus the heritability estimate of the log-transformed relative abundance of each ORF. These estimates were obtained using a univariate animal model in ASReml v4.2 [48] and ranged from 0 to 0.78 with a mean of 0.07 and median 0.04. The heritability estimates were assumed to be known without error. The order of diagonal elements was such that it matched the order of ORF in $\mathbf{S}$. Then weighted MRM were created by including $\mathbf{D}$ in the manner as shown below for the M1 and M2 construction methods, Eq. (6) and Eq. (7) respectively:

6 $${\mathbf{M}}_{{1{\text{W}}}} = \frac{{\left( {{\mathbf{S}} - {\mathbf{N}}} \right){\mathbf{D}}\left( {{\mathbf{S}} - {\mathbf{N}}} \right){^{\prime}}}}{{\mathop \sum \nolimits_{{{\text{j}} = 1}}^{{\text{m}}} {\text{var}}\left( {{\text{s}}_{{\text{j}}} } \right)}},$$

7 $${\mathbf{M}}_{{2{\text{W}}}} = \frac{1}{{\text{m}}}{\mathbf{CDC^{\prime}}}.$$

The data-driven adjustment was inspired by the multi-population GRM methodology created to accommodate data from animals with differing genetic backgrounds in the same evaluation [49, 50]. Here, the animals were split into different groups based on their metagenome composition. Rather than imposing grouping based on presumed differences, such as diet, metagenome data were used to group animals, hence data-driven. First, a principal component analysis was performed on the ORF using the FactoMineR package [51] (See Additional file 1, Figure S1). Next, the animals were sorted into two groups based on their values for the first four principal components using k-means clustering in the eclust function from the factoextra package [52]. The value k = 2 was determined to be the most suitable number of clusters based on the consensus of 24 indexes using Euclidean distancing as calculated by the NBClust function in the NBClust package [53]. The two groups primarily delineated animals by sex, or, in terms of diet, primarily concentrate and forage; however, there were 19 steers that clustered with the heifers. Once the animals were assigned into different groups, the centering and scaling of their metagenome profiles were done based on the mean and standard deviation of that group. Following the description of a multi-population $\mathbf{G}$ matrix [50], the multi-population ${\mathbf{M}}_{\text{P}}$ matrix was constructed as follows:

Using M1 in Eq. (8):

8 $${\mathbf{M}}_{{1{\text{N}}}} = \left[ {\begin{array}{*{20}c} {\frac{{\left( {{\mathbf{S}}_{1} - {\mathbf{N}}_{1} } \right)\left( {{\mathbf{S}}_{1} - {\mathbf{N}}_{1} } \right){\prime} }}{{\mathop \sum \nolimits_{{{\text{j}} = 1}}^{{\text{m}}} {\text{var}}\left( {{\text{s}}_{{1{\text{j}}}} } \right)}}} & {\frac{{\left( {{\mathbf{S}}_{1} - {\mathbf{N}}_{1} } \right)\left( {{\mathbf{S}}_{2} - {\mathbf{N}}_{2} } \right){\prime} }}{{\sqrt {\mathop \sum \nolimits_{{{\text{j}} = 1}}^{{\text{m}}} {\text{var}}\left( {{\text{s}}_{{1{\text{j}}}} } \right)} \sqrt {\mathop \sum \nolimits_{{{\text{j}} = 1}}^{{\text{m}}} {\text{var}}\left( {{\text{s}}_{{2{\text{j}}}} } \right)} }}} \\ {\frac{{\left( {{\mathbf{S}}_{2} - {\mathbf{N}}_{2} } \right)\left( {{\mathbf{S}}_{1} - {\mathbf{N}}_{1} } \right){\prime} }}{{\sqrt {\mathop \sum \nolimits_{{{\text{j}} = 1}}^{{\text{m}}} {\text{var}}\left( {{\text{s}}_{{2{\text{j}}}} } \right)} \sqrt {\mathop \sum \nolimits_{{{\text{j}} = 1}}^{{\text{m}}} {\text{var}}\left( {{\text{s}}_{{1{\text{j}}}} } \right)} }}} & {\frac{{\left( {{\mathbf{S}}_{2} - {\text{N}}_{2} } \right)\left( {{\mathbf{S}}_{2} - {\mathbf{N}}_{2} } \right){\prime} }}{{\mathop \sum \nolimits_{{{\text{j}} = 1}}^{{\text{m}}} {\text{var}}\left( {{\text{s}}_{{2{\text{j}}}} } \right)}}} \\ \end{array} } \right],$$

where all terms are as defined previously.

Using M2, the centering and scaling where applied the $\mathbf{C}$ matrices before taking the cross-product such that elements of matrix $\mathbf{C}$ for cluster $\text{c}$ (${\mathbf{C}}_{\text{c}}$) in Eq. (9):

9 $${\mathbf{C}}_{{{\text{ijc}}}} = \frac{{{\mathbf{S}}_{{{\text{ijc}}}} - \overline{{{\mathbf{S}}_{{.{\text{jc}}}} }} }}{{\sqrt {{\text{var}}\left( {{\mathbf{S}}_{{.{\text{jc}}}} } \right)} }},$$

where the value of ORF $j$ for animal $i$ in cluster $\text{c}$ was centered and scaled by the mean and variance of ORF $j$ in cluster $c$. The completed MRM described in Eq. (10):

10 $${\mathbf{M}}_{{2{\text{P}}}} = \left[ {\begin{array}{*{20}c} {\frac{1}{{\text{m}}}{\mathbf{C}}_{1} {\mathbf{C}}_{1} ^{\prime}} & {\frac{1}{{\text{m}}}{\mathbf{C}}_{1} {\mathbf{C}}_{2} ^{\prime}} \\ {\frac{1}{{\text{m}}}{\mathbf{C}}_{2} {\mathbf{C}}_{1} ^{\prime}} & {\frac{1}{{\text{m}}}{\mathbf{C}}_{2} {\mathbf{C}}_{2} ^{\prime}} \\ \end{array} } \right].$$

Each MRM, regardless of adjustment, was tuned with a diagonal matrix with elements of 0.001 (${\mathbf{M}}_{\text{T}}$ = $\mathbf{M}$ + 0.001$\mathbf{I}$) to allow for inversion.

Jarquín et al. [54] established that the interaction between two random effects can be accounted for through the Hadamard product of the (co)variance matrices of the two effects. This element-wise product technique to capture the host-microbiome interaction has been reported by others [16, 20]. Thus, the interaction of $\mathbf{G}$ and $\mathbf{M}$, $\mathbf{J}$, described in Eq. (11):

11 $${\mathbf{J}} = {\mathbf{G}} \circ {\mathbf{M}}.\user2{ }$$

Each method-adjustment combination of the untuned $\mathbf{M}$ was multiplied by untuned $\mathbf{G}$ and yielded a corresponding $\mathbf{J}$, resulting in six unique interaction (co)variance matrices. Each interaction (co)variance matrix was tuned with a diagonal matrix with elements of 0.001 (${\mathbf{J}}_{\mathbf{T}}$ = $\mathbf{J}$ + 0.001$\mathbf{I}$).

### Variance component and parameter estimation

The models fit were univariate animal models with either ADDMI or ADG as the phenotype of interest. All models had the same fixed effects including expected heterozygosity and breed proportions (linear covariates) and management group (16 levels). There were three degrees of complexity for the models: singular, joint, and interaction. Singular models only included one random effect, either the genomic or metagenomic effect. Joint models included both genomic and metagenomic effects. Lastly, interaction models included a genomic effect, a metagenomic effect, and an interaction effect. In total, 38 models were fitted: the host genomic model, 6 singular metagenomic models (one for each method-adjustment combination), 6 joint models, and 6 interaction models for each of the two phenotypes of interest.

The host genomic model was defined in Eq. (12):

12 $${\mathbf{y}} = {\mathbf{X\beta }} + {\mathbf{Zu}} + {\mathbf{e}},$$

where $\mathbf{y}$ is a vector of phenotypes, $\mathbf{X}$ and $\mathbf{Z}$ are incidence matrices, ${\varvec{\upbeta}}$ is vector of fixed effects, **u** is a vector of random animal genetic effects assumed to be distributed as $\text{N}\left(0,{\mathbf{G}}_{\text{T}}{\upsigma }_{\text{u}}^{2}\right)$ where ${\mathbf{G}}_{\text{T}}$ is the GRM defined above and ${\upsigma }_{\text{u}}^{2}$ is the additive genetic variance, and $\mathbf{e}$ is a vector of random effects assumed to be distributed $\text{N}\left(0,\mathbf{I}{\upsigma }_{\text{e}}^{2}\right)$ where $\mathbf{I}$ is the identity matrix and ${\upsigma }_{\text{e}}^{2}$ is the residual variance.

Similarly, the singular metagenomic models were defined in Eq. (13):

13 $${\mathbf{y}} = {\mathbf{X\beta }} + {\mathbf{Wm}} + {\mathbf{e}},$$

where $\mathbf{W}$ is an incidence matrix, $\mathbf{m}$ is a vector of random metagenome effects assumed to be distributed as $\text{N}\left(0,{\mathbf{M}}_{\text{T}}{\upsigma }_{\text{m}}^{2}\right)$ where ${\mathbf{M}}_{\text{T}}$ is one of the method-adjustment combinations of MRM defined above and ${\upsigma }_{\text{m}}^{2}$ is the microbial variance, and all other terms are as defined previously.

The joint models were simply the genetic and metagenome effects together (Eq. (14)):

14 $${\mathbf{y}} = {\mathbf{X\beta }} + {\mathbf{Zu}} + {\mathbf{Wm}} + {\mathbf{e}},{ }$$

where all terms are as defined previously.

Finally, the interaction models include an interaction effect (Eq. (15)):

15 $${\mathbf{y}} = {\mathbf{X\beta }} + {\mathbf{Zu}} + {\mathbf{Wm}} + {\mathbf{To}} + {\mathbf{e}},{ }$$

where $\mathbf{T}$ is an incidence matrix and ${\varvec{o}}$ is a vector of random interaction effects assumed to be distributed as $\text{N}\left(0,{\mathbf{J}}_{\text{T}}{\upsigma }_{\text{o}}^{2}\right)$ where ${\mathbf{J}}_{\text{T}}$ is the genome-metagenome interaction matrix defined above and ${\upsigma }_{\text{o}}^{2}$ is the interaction variance, and all other terms are as defined above. Importantly, ${\mathbf{J}}_{\text{T}}$, $\mathbf{T}$, and $\mathbf{o}$ were only included if both the genomic and metagenomic effects were also included.

Heritability (${h}^{2}$) was defined as the ratio of additive genetic variance to the sum of all variances. The proportion of variation explained by the metagenome effect (*m*^*2*^) was defined as the ratio of the metagenomic variance to the sum of all variance estimates in a given model. The sum of the host genomic and metagenomic variance (${i}_{a}^{2}$) was defined as the ratio of the sum of the additive genetic variance and metagenomic variance to the sum of all variance estimates in a given model. The interaction of host genotype and metagenome effects (${i}_{i}^{2}$) was defined as the ratio of the sum of the additive genetic variance, metagenomic variance, and interaction variance to the sum of all variance estimates in a given model. The variance components and Akaike Information Criterion (AIC) were taken from the results of ASReml v4.2 [48] for models provided the complete dataset. For AIC, lower values are more desirable.

### Phenotypic prediction

Given the structure of the data, two forms of validation were employed, a leave-one-diet-out (LODO) and fourfold cross-validation (F). The LODO strategy involved training on three diets and testing on the fourth. Given there were two forage-based diets and two-concentrate based diets, the diet type being predicted was represented by 1/3 of the training data. This was chosen because the 4 diets represented a natural split of the data. For the fourfold cross-validation, animals were randomly allocated to one of four folds. The category of sex, here a proxy for forage or concentrate focused diet, was balanced between the folds. When testing, 75% of the data (3 groups) were used for training and the other 25% used for testing. These folds were replicated 5 times, resulting in a total of 20 results per model for this strategy. The number of folds was chosen to better mimic the LODO strategy so that each fold, regardless of strategy, would be trained on about 75% of the data. This strategy was intended to set a benchmark for prediction accuracy. Given the two cross-validation approaches there were four sets of results: ADMMI-LODO, ADDMI-F, ADG-LODO, and ADG-F.

Adjusted phenotypes were calculated as ${\mathbf{y}}_{\text{adj}}=\mathbf{y}-\mathbf{X}\widehat{{\varvec{\upbeta}}}$. Accuracy was calculated as the Pearson correlation between the adjusted phenotypes and the sum of random effect solutions for each animal in the test data (termed the total animal merit (TAM)). Further, the correlation between the adjusted phenotypes and each individual random effect solution was also calculated. These were the estimated breeding value (EBV) for solutions of the host genomic effect, the estimated metagenome value (EMV) for solutions of the metagenome effect, and the estimated interaction value (EIV) for solutions of the interaction effect. For models with only one random effect fitted (i.e., host genomic or metagenomic) the total animal effect was equivalent to one of the random effect solutions (e.g., TAM = EBV for the host genomic only model). To assess the departure of the prediction from the adjusted phenotype, a root mean square error (RMSE) value was calculated. Additionally, the adjusted phenotype was regressed on the TAM of each model. The coefficient of that regression was treated as a dispersion parameter. For dispersion, values closer 1 are more desirable. All adjusted phenotypes and model metrics were performed in R v. 4.4 [55] using output from ASReml v. 4.2 [48] (Table 1).

## Results

### Variance component estimation and model fit criteria

The ratios of variance components for ADDMI and ADG are in Tables 2 and 3, respectively. Variance component estimates can be found in Additional File 2, S1-S6. The ${h}^{2}$ estimates from the genomic only model were 0.33 for ADDMI and 0.18 for ADG. The ${h}^{2}$ estimates for the more complex models tend to decrease from the ${h}^{2}$ estimate of the genomic model with larger differences observed for ADG than for ADDMI.

**Table 2:** Parameter estimates (standard errors) for average daily dry matter intake (ADDMI)

Method^a^	Adjustment	Complexity	${h}^{2}$ ^b^	${m}^{2}$ ^c^	${i}_{a}^{2}$ ^d^	${i}_{i}^{2}$ ^e^	AIC^f^
Genomic	Naïve	Singular	0.33 (0.13)				1146
Method 1	Data-driven	Singular		0.10 (0.06)			2150
Method 2	Data-driven	Singular		0.08 (0.06)			1972
Method 1	Data-driven	Joint	0.32 (0.12)	0.11 (0.06)	0.43 (0.13)		2261
Method 2	Data-driven	Joint	0.32 (0.13)	0.09 (0.06)	0.41 (0.13)		2082
Method 1	Data-driven	Interaction	0.32 (0.12)	0.11 (0.06)	0.43 (0.13)	0.43 (0.13)	2382
Method 2	Data-driven	Interaction	0.32 (0.13)	0.09 (0.06)	0.41 (0.13)	0.41 (0.13)	2268
Method 1	Naïve	Singular		0.25 (0.11)			2998
Method 2	Naïve	Singular		0.16 (0.08)			2241
Method 1	Naïve	Joint	0.26 (0.11)	0.26 (0.11)	0.52 (0.13)		3108
Method 2	Naïve	Joint	0.29 (0.12)	0.16 (0.07)	0.45 (0.13)		2352
Method 1	Naïve	Interaction	0.25 (0.11)	0.23 (0.11)	0.48 (0.13)	0.57 (0.14)	3186
Method 2	Naïve	Interaction	0.29 (0.12)	0.16 (0.08)	0.45 (0.13)	0.47 (0.14)	2407
Method 1	Weighted	Singular		0.27 (0.11)			3054
Method 2	Weighted	Singular		0.17 (0.08)			2298
Method 1	Weighted	Joint	0.25 (0.11)	0.27 (0.11)	0.53 (0.13)		3165
Method 2	Weighted	Joint	0.29 (0.12)	0.18 (0.08)	0.46 (0.13)		2409
Method 1	Weighted	Interaction	0.24 (0.11)	0.25 (0.11)	0.49 (0.13)	0.58 (0.14)	3278
Method 2	Weighted	Interaction	0.28 (0.12)	0.17 (0.08)	0.45 (0.13)	0.48 (0.14)	2513

Parameter estimates (standard errors) for average daily dry matter intake (ADDMI) calculated from a variety of methods, adjustments, and complexities to model the random effects

^a^Method 1 denotes the model that used a microbial (co)variance matrix created according to Van Raden [44] method 1. Method 2 denotes the model microbial (co)variance matrix created according to Ross et al. [17]

^b^Ratio of additive genetic variance divided by the sum of the additive genetic variance and the residual variance

^c^Ratio of microbial variance divided by the sum of all variances

^d^Ratio of sum of additive genetic and microbial variances divided by the sum of all variances

^e^Ratio of sum of additive genetic, microbial, and interaction variances divided by the sum of all variances

^f^Akaike Information Criterion

**Table 3:** Parameter estimates (standard errors) for average daily gain (ADG)

Method^a^	Adjustment	Complexity	${h}^{2}$ ^b^	${m}^{2}$ ^c^	${i}_{a}^{2}$ ^d^	${i}_{i}^{2}$ ^e^	AIC^f^
Genomic	Naïve	Singular	0.18 (0.13)				−1135
Method 1	Data-driven	Singular		0 (0)			−309
Method 2	Data-driven	Singular		0 (0)			−129
Method 1	Data-driven	Joint	0.18 (0.13)	0 (0)	0.18 (0.13)		−12
Method 2	Data-driven	Joint	0.18 (0.13)	0 (0)	0.18 (0.13)		−193
Method 1	Data-driven	Interaction	0.18 (0.12)	0.11 (0.07)	0.30 (0.13)	0.56 (0.15)	103
Method 2	Data-driven	Interaction	0.20 (0.12)	0 (0)	0.20 (0.12)	0.50 (0.16)	−13
Method 1	Naïve	Singular		0.49 (0.12)			718
Method 2	Naïve	Singular		0.37 (0.11)			−42
Method 1	Naïve	Joint	0.11 (0.08)	0.50 (0.12)	0.60 (0.12)		834
Method 2	Naïve	Joint	0.15 (0.10)	0.37 (0.10)	0.52 (0.13)		73
Method 1	Naïve	Interaction	0.11 (0.08)	0.50 (0.12)	0.60 (0.12)	0.67 (0.12)	910
Method 2	Naïve	Interaction	0.14 (0.10)	0.30 (0.11)	0.45 (0.13)	0.82 (0.14)	116
Method 1	Weighted	Singular		0.50 (0.13)			775
Method 2	Weighted	Singular		0.39 (0.11)			15
Method 1	Weighted	Joint	0.11 (0.08)	0.51 (0.12)	0.61 (0.12)		890
Method 2	Weighted	Joint	0.15 (0.09)	0.39 (0.11)	0.54 (0.13)		130
Method 1	Weighted	Interaction	0.11 (0.07)	0.51 (0.12)	0.61 (0.12)	0.68 (0.12)	1003
Method 2	Weighted	Interaction	0.14 (0.09)	0.31 (0.11)	0.45 (0.13)	0.83 (0.14)	223

Parameter estimates (standard errors) for average daily gain (ADG) calculated from a variety of methods, adjustments, and complexities to model the random effects

^a^Method 1 denotes the model that used a microbial (co)variance matrix created according to Van Raden [44] method 1. Method 2 denotes the model that uses a microbial (co)variance matrix created according to Ross et al. [17]

^b^Ratio of additive genetic variance divided by the sum of the additive genetic variance and the residual variance

^c^Ratio of microbial variance divided by the sum of all variances

^d^Ratio of sum of additive genetic and microbial variances divided by the sum of all variances

^e^Ratio of sum of additive genetic, microbial, and interaction variances divided by the sum of all variances

^f^Akaike Information Criterion

The *m*^*2*^ estimates from the singular metagenomic models differed by adjustment. The models with a data-driven ${\mathbf{M}}_{\text{T}}$ resulted in lower *m*^*2*^ estimates than those models with naïve or weighted ${\mathbf{M}}_{\text{T}}$. In fact, the singular microbial models with a data-driven ${\mathbf{M}}_{\text{T}}$ had a *m*^*2*^ estimate of zero for ADG. The singular microbial models which included a M1 ${\mathbf{M}}_{\text{T}}$ regularly captured more variation than the models using M2.

As would be expected, more complex models captured a greater proportion of variation. The greatest increases were usually in going from single random effect models to joint models. Adding an interaction term to the model usually increased the proportion of variation captured by the random effects. For ADDMI, the increase in variation explained by the model depended on the method used to form ${\mathbf{M}}_{\text{T}}$. The exception to this were models with a data-driven ${\mathbf{M}}_{\text{T}}$ where the interaction term did not explain more variation compared to the joint model. The interaction models with ${\mathbf{M}}_{\text{T}}$ formed by M2 that were either naïve or weighted often had the same proportion of variation explained, or slightly more, than their joint counterparts. The interaction models with a M1 ${\mathbf{M}}_{\text{T}}$ that were either naïve or weighted resulted in ${i}_{i}^{2}$ estimates approximately 0.10 greater than ${i}_{a}^{2}$ produced from the joint models with the same adjustments to ${\mathbf{M}}_{\text{T}}$. In comparison, the interaction models demonstrated a higher proportion of variation explained compared to their joint complements for ADG, regardless of method or adjustment. In particular, the percentage of variation explained by the sum of the random effect variances from the joint models with a M2 ${\mathbf{M}}_{\text{T}}$ that was either naïve or weighted was 45%; whereas the amount of variation explained by the sum of the random effect variances by those models with the same ${\mathbf{M}}_{\text{T}}$ but with the addition of an interaction term was greater than 80%.

The Akaike Information Criterion (AIC) is reported in Tables 2 and 3. The genomic only models had the lowest AIC, followed by those models with the data-driven adjusted ${\mathbf{M}}_{\text{T}}$. Models with an ${\mathbf{M}}_{\text{T}}$ created by M2 had lower AIC values than models with a M1 ${\mathbf{M}}_{\text{T}}$ when the other factors of the model were held constant.

### Prediction metrics

The complexity of some models resulted in failure to converge. Specifically, these models were from the fourfold cross validation set when ADG was the phenotype of interest. One fold with an interaction model with a naïve M2 ${\mathbf{M}}_{\text{T}}$ and three folds with interaction models with weighted M2 ${\mathbf{M}}_{\text{T}}$ had this issue. These models were omitted from the results.

**Fig. 2:**
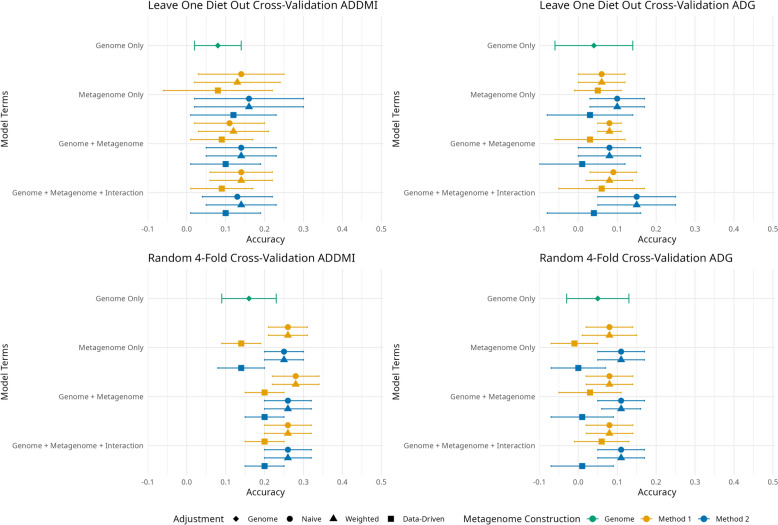
Median and standard deviation of correlation between sum of random effects and adjusted phenotype for average daily dry matter intake (ADDMI) and average daily gain (ADG) using different prediction model and two forms of cross-validation, a random fourfold strategy (fourfold) and splitting the data based on ration formulation (Leave-One-Diet-Out)

In general, across both traits and both cross-validation strategies, the most accurate models were those with the attributes of being either an interaction or joint model with an ${\mathbf{M}}_{\text{T}}$ constructed using M2 with either a naïve or weighted adjustment, although differences among models were numerically slight (Pearson correlation differences of 0.01–0.03). The exception was ADDMI-F. In this case, the interaction and joint models with either a naïve or weighted ${\mathbf{M}}_{\text{T}}$ formed using M1 were slightly more accurate. Figure 2 depicts these differences, and full details are in Additional File 3, S7-S8 & S13-S14.

Prediction accuracy as discussed is relative to the TAM of each model. Of the single random effect models, the genomic only model often resulted in accuracies that were similar to the singular metagenomic effect models. For some trait/cross-validation combinations, however, the genomic models did not do as well. For example, the host genomic model for ADDMI-F, despite being the most accurate genomic model for the trait/cross-validation combinations, was most similar in terms of prediction accuracy compared to the singular metagenomic effect models that had a data-driven ${\mathbf{M}}_{\text{T}}$, but was outperformed by the metagenomic models with a naïve or weighted ${\mathbf{M}}_{\text{T}}$. Among the metagenomic effect only models, the differences in accuracy between models with naïve and weighted models were minimal when all other attributes were matched. Within the data-driven ${\mathbf{M}}_{\text{T}}$ models, M1 and M2 were similar in TAM accuracy, regardless of trait/cross-validation combination. However, for singular random effect models with naïve or weighted ${\mathbf{M}}_{\text{T}}$, the method of constructing ${\mathbf{M}}_{\text{T}}$ did have an impact on accuracy depending on the trait/cross validation combination. For most cross-validations, ${\mathbf{M}}_{\text{T}}$ formed with method 2 with either the naïve or weighted adjustment resulted in a more accurate predictions than method 1 with the same adjustment, albeit minimally (Pearson correlation differences of 0.03–0.05). The exception was for ADG-F where the M1 with a naïve or weighted adjustments slightly outperformed the M2.

Joint models were always more accurate than singular metagenomic effect models when the same ${\mathbf{M}}_{\text{T}}$ was used. Joint models have three random effects for which prediction accuracy was estimated: TAM, EBV, and EMV (full details in Additional File 3 S7-S8 & S13-S14). Within a trait/cross-validation combination, the accuracy of the EBV was fairly static between model complexities. The differences in TAM reported herein were driven by differences in EMV. The results of the joint models followed the results from the singular microbial effect models. In general, the models with a data-driven ${\mathbf{M}}_{\text{T}}$ were less accurate than those with a naïve or weighted ${\mathbf{M}}_{\text{T}}$, irrespective of construction method. Comparing the construction methods within adjustment revealed that M2 often led to a more accurate TAM, though the magnitude of the advantage depended on the other attributes of the model. The ADDMI-F validation was the exception where M1 yielded marginally more accurate TAM.

The additional accuracy gained by adding an interaction term varied by trait/cross-validation combination, construction method, and adjustment. In most cases, the prediction accuracy of the TAM of interaction models was the same or marginally greater than the joint models when matched for all other model attributes. Overall, models which used a data-driven ${\mathbf{M}}_{\text{T}}$ were less accurate than those with a naïve or weighted ${\mathbf{M}}_{\text{T}}$. Models with a M2 ${\mathbf{M}}_{\text{T}}$ were slightly more accurate than those with a M1 ${\mathbf{M}}_{\text{T}}$ with the exception of ADDMI-F.

Comparisons of the accuracy from each random effect from each cross-validation strategy was trait dependent. Using TAM as the predictor, the fourfold strategy was more accurate than the LODO strategy for ADDMI, but the two strategies had comparable accuracy for ADG. Conversely, the fourfold strategy always had greater accuracy when EBV were used. Predicting adjusted phenotype using EMV resulted in greater accuracy for the fourfold strategy compared to the LODO strategy, but only when the trait of interest was ADDMI. Lastly, EIV based predictions had the same pattern as TAM based predictions where the predictions were more accurate in the fourfold strategy for ADDMI, but equivalent between the two cross-validation strategies for ADG.

Other model metrics elucidated further findings. The RMSE was minimally different among models within a given trait/cross-validation strategy. Differences for this metric were around 0.01 and are not shown in detail.

**Fig. 3:**
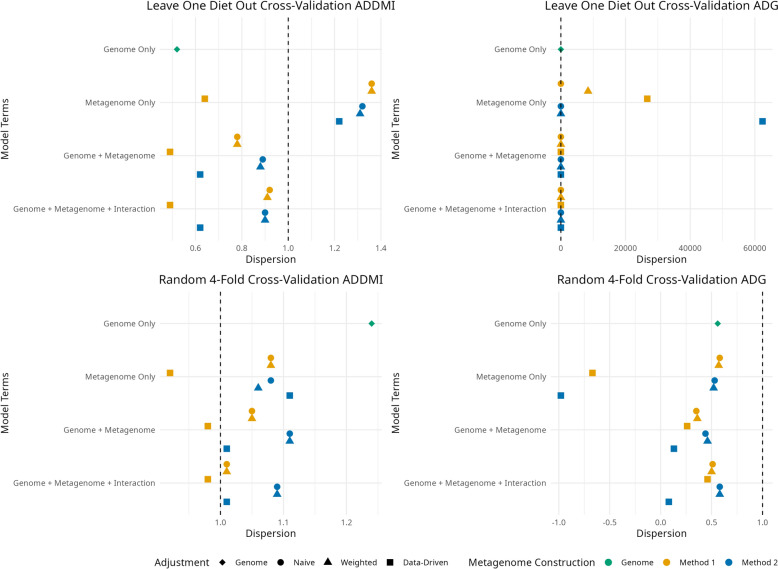
Median dispersion value between sum of random effects and adjusted phenotype for average daily dry matter intake (ADDMI) and average daily gain (ADG) using different prediction model and two forms of cross-validation, a random fourfold strategy (fourfold) and splitting the data based on ration formulation (Leave-One-Diet-Out)

Median dispersion parameters (Fig. 3) ranged greatly. Extreme values were noted for three models in the LODO strategy for ADG. These models only contained the metagenome effect and included either a weighted M1 ${\mathbf{M}}_{\text{T}}$ or a data-driven ${\mathbf{M}}_{\text{T}}$ created using either method. These values resulted from folds where the random effect variance converged to zero. In general, models with multiple random effects, i.e. joint or interaction models, had less dispersion than models with a single random effect. Those models which used a naïve or weighted ${\mathbf{M}}_{\text{T}}$ also tended to be less dispersed than models with a data-driven MRM when the other features of the models were held constant.

## Discussion

### Variance components, parameter estimation, and model fit criteria

While the heritability estimates from the host genomic effect only model for ADDMI and ADG were within the range previously reported by others [3, 4], it is worth noting they are lower than is commonly reported. This likely impacted the prediction accuracy for this trait. The reduction in additive genetic variance (Additional Files 2 Table S1, S2) when the metagenomic effect was fitted was expected for the naïve models given the influence of host genotype on rumen metagenome composition [11, 26, 27]. An argument can be made that the loss of additive genetic variance is due to the metagenome effect absorbing part of the host genetic effect which is mediated through the rumen metagenome. Khanal et al. [16], Saborío-Montero et al. [20], and Hess et al. [25], all reported a decrease in heritability estimates when microbial effects were fitted. Given a host’s genome affects its metagenome which, in turn, affects the phenotype (genomic effects mediated through the metagenome), it is possible the MRM would absorb variance which should be attributed to the genomic effect. In other words, the proportion of variation attributed to the host genomic effect mediated through the metagenome is the difference between heritability in the genomics only models and the heritability in the joint models. The weighted adjustment applied to $\mathbf{M}$ was intended to curb this issue. By down-weighting the ORF which are influenced by host genetics, the metagenome-mediated genomic effects should be captured by the random genomic effect alone. However, the median heritability of the ORF themselves was 0.04 and 69% of the 16,583 estimates were below 0.10. Consequently, the weighted ${\mathbf{M}}_{\mathbf{T}}$ contained information that was very similar to their naïve counterparts. Therefore, it was unsurprising to see a high degree of similarity between the models with a naïve adjusted ${\mathbf{M}}_{\mathbf{T}}$ and weighted adjusted ${\mathbf{M}}_{\mathbf{T}}$. Importantly, the weightings applied herein assumed that the estimates of heritability used were known without error. Interestingly the models that included the data-driven ${\mathbf{M}}_{\mathbf{T}}$ seemed to have similar estimates of ${h}^{2}$ as the genomic only models. Moreover, the models that included data-driven ${\mathbf{M}}_{\mathbf{T}}$ captured less variation overall, likely due to minimal information sharing between the two partitions of ${\mathbf{M}}_{\mathbf{T}}$. Microbiability estimates from data-driven models were mostly zero for ADG, but the *m*^*2*^ estimates from the other adjustments were moderate to high. Therefore, the microbial relationships between the two clusters seemed to be important for capturing variation among animals. The results between construction methods showed a much clearer picture for ${m}^{2}$. Comparatively, M1 and M2 resulted in highly correlated matrices although individual elements varied. For these data, the M1 models had greater estimates of ${m}^{2}$.

The single random effect models established that both host genetics and the metagenome explain at least some variation for both traits. Thus, it follows the joint models, with both effects included, explained more total variation than any single random effect model. Given the ratio of additive genetic variance to the residual variance increases in models with multiple random effects, although sometimes slight, it would be expected that the model derived accuracy of EBV would also increase. This has been observed by others when prediction has included both host genomic and microbiome data wherein EBV accuracy can be increased by up to 22% [11].

The influence of the interaction effect seemed model and trait dependent. Previous literature has shown the genome-by-microbiome interaction to increase the amount of variation explained [16]; however, that increase can be to varying degrees. Saborío-Montero et al. [20] reported adding an interaction term only increased the proportion of variance explained by 0.01–0.15 compared to joint models. A similar result was found in the current study, but with a greater range in the increase of ${i}_{i}^{2}$ over ${i}_{a}^{2}$, especially for ADG. Additionally, M1 models had increased estimates of ${i}_{a}^{2}$ and ${i}_{i}^{2}$ compared to M2. However, it is possible that the variation explained by the interaction fitted herein was inflated due to the increased model rank.

While the AIC is often the lowest for genomic only or simpler models, more complex models often had greater accuracy of prediction. The goal of AIC is to balance model fit with complexity, so it is unsurprising that less complex models have lower AIC given the size of the dataset used. It is apparent for these results that there is a trade-off between model complexity and prediction accuracy in validation.

### Prediction metrics

Within a trait/cross-validation strategy combination, the difference in correlations between the most accurate and least accurate model were approximately 0.10. Top performing models contained more than one random effect and did not use a data-driven adjustment.

Predictive ability of the host genomics only models averaged over cross-validation methods was greater for ADDMI than for ADG. This would be expected given the greater heritability estimate for ADDMI. However, the genomics only model never outperformed all the microbiome effect only models when the naïve or weighted adjusted ${\mathbf{M}}_{\text{T}}$ where used. Given ADDMI and ADG are traits related to rumen function and the rumen microbiome, it is logical that the metagenome effect is predictive of phenotype. Khanal et al. [16] similarly reported a microbiome effect only model was more predictive than a genomic effect only model for a few traits including carcass average daily gain and belly weight in swine. Between the metagenome effect only models, the models with a data-driven ${\mathbf{M}}_{\text{T}}$ were less accurate than models with either a naïve or weighted ${\mathbf{M}}_{\text{T}}$. An adjustment similar to the data-driven adjustment used herein was performed by Hess et al. [25]. Those authors standardized microbiome profile by cohort (a combination of age group with either grass-based or lucerne (alfalfa) pellet-based diet) then took the transpose of the standardized matrix and calculated the correlation of the microbial data between each pair of animals. The authors also created an MRM using the same method, but with microbiome data standardized by the whole dataset. Interestingly, Hess et al. [25] showed little difference in the phenotypic prediction accuracy between the standardization protocols. This contrasts with the results from this study as the models with a data-driven ${\mathbf{M}}_{\text{T}}$ clearly yielded the lowest accuracies, albeit the differences between the adjustments varied depending on the other attributes of the models. In the current study the fixed effect of management group, that included diet and sex, was included in the model. The fact that diet, indirectly, was fitted as a fixed effect and (also indirectly) used to partition the two sub-populations in ${\mathbf{M}}_{\text{D}}$ contributed to the performance of the models that included a data-driven ${\mathbf{M}}_{\text{T}}$. Additionally, research with SNP data has shown that GRM constructed using SNP with differing MAF cutoffs can result in differing relationship estimates and differing EBV [56]. This is roughly analogous to the filtering threshold for the ORF used to create the MRM. The thresholds used here reduced the original data of 43 million ORF to 16,583 ORF and excluded any rare or lowly abundant ORF. However, including those ORF may make a change in the MRM and the prediction accuracy. This result may be especially true with the data-driven adjustment given how it incorporates the differences between diet types. This difference may be exacerbated by the difference in primary energy source (grain vs forage) but also by the fact that the steers received monensin which is known to alter the composition of the rumen microbiome [57, 58]. Another avenue to explore might be to treat the trait of the two clusters (proxy for diet) as different traits in a bivariate model [50]. Instead of forcing a single metagenomic variance, a bivariate approach allows each cluster to have its own variance with a covariance between clusters.

More complex models, the joint and interaction models, having greater prediction accuracy over a model with a single random effect is similar to the finding of Khanal et al. [16] and Saborío-Montero et al. [20]. The authors of those studies concluded that an interaction term should be included in the model. However, both showed that adding an interaction only resulted in a marginal increase in accuracy over an joint model. In the current study the TAM accuracy was only increased minimally compared to the joint models. For large datasets, the increase in accuracy of an interaction model needs to be weighed against the additional time and computing power required to create and invert a third covariance matrix.

Interestingly, differences in accuracy were more dependent on the trait evaluated and less so on the cross-validation strategy employed. More specifically, the ADDMI-F cross-validation seemed to behave differently than the other trait/cross-validation combinations. The highest median accuracy was for ADDMI-F at 0.28 whereas the other cross-validations consistently had maximum median accuracies of 0.11–0.16. There was likely a combination of factors that increased the accuracy for this scenario. One such factor might be that data from HD2 was included in all folds of ADDMI-F. Given that HD2 was the most difficult diet to predict in ADDMI-LODO dataset (See Additional Files 3, S7 & S13), this may imply that ration differences influence differences in accuracy between the two ADDMI datasets.

### Considerations for practical implementation

To perform phenotype predictions at a scale where they can have a discernable effect on profitability requires certain considerations as to the infrastructure for such an enterprise. Firstly, samples need to be collected. The method of sample collection should be something which is easy to perform and works at the speed of commerce. While the data presented herein includes metagenomic information from the rumen, esophageal tubing may not be the most efficient choice. An oral or rectal swab would be more convenient. Moreover, these types of swabs, those in close contact with the animal’s own tissue, open the possibility to impute the host’s genotype from reads generated at the same time as the metagenomic data [59], but this host DNA also may raise the cost of microbial sequencing due to more redundant (host) sequence. Timing is also a consideration. The microbiome fluctuates over time [46] and that temporal shift can affect prediction accuracy [15]. Moreover, with respect to oral swabs, the sample collection time in relation to eating and rumination events could add additional complexity. Setting a specific recommended time for sample collection (e.g., at weaning) may not maximize prediction accuracy for all traits in consideration but could optimize accuracy, sample uniformity, and producer convenience. Alternately serial samples, aligned in time with when phenotypes of interest would be expressed, would be an alternative. Commercialization of metagenomic sequencing, generating predictions, and delivery of predictions in a timely fashion to take management decisions would also be critical for technology adoption.

## Conclusions

Host genetics, rumen metagenome composition, and genotype-metagenome interaction all have an impact on production traits in beef cattle. The amount of variation captured is a function of trait, how the effect of the metagenome is modeled, and the level of complexity of the model. In general, models with a metagenome (co)variance matrix formed by dividing by the square root of the sum of the variances of each ORF (method 1) captured more variation than models that scaled each ORF by its standard deviation directly (method 2). Moreover, including both the host genotype and metagenome effects in the model captured more variation than either alone. Further variation was explained when the interaction effect was also included.

Phenotype prediction metrics also varied by trait, modeling of the metagenome effect, and level of complexity of the model. While no one combination was consistently the most accurate, multiple random effects consistently outperformed in terms of both accuracy and dispersion than the fitting of only the genomic or only the metagenome effect.

## Supplementary Information

Additional file 1. Figure S1. Principal Component Analysis of Rumen Metagenome by Diet.

Additional file 2. Table S1. Variance component estimates for average daily dry matter intake (ADDMI) using host genomic and rumen metagenomic data. Table S2. Variance component estimates for average daily gain (ADG) using host genomic and rumen metagenomic data. Table S3. Median prediction accuracy (standard deviation) of 4-fold validation for average daily dry matter intake (ADDMI). Table S4. Median prediction accuracy (standard deviation) of 4-fold validation for average daily gain (ADG). Table S5. Median prediction accuracy (standard deviation) of leave-one-out validation for average daily dry matter intake (ADDMI). Table S6. Median prediction accuracy (standard deviation) of leave-one-out validation for average daily gain (ADG).

Additional file 3. Table S7. Prediction Accuracy of Each Component of Each Fold for Average Daily Dry Matter Intake from the 4-fold Cross-Validation Strategy. Table S8. Prediction Accuracy of Each Component of Each Fold for Average Daily Gain from the 4-fold Cross-Validation Strategy. Table S9. Root Mean Square Error of Each Fold for Average Daily Dry Matter Intake from the 4-fold Cross-Validation Strategy. Table S10. Root Mean Square Error of Each Fold for Average Daily Gain from the 4-fold Cross-Validation Strategy. Table S11. Dispersion Value of Each Fold for Average Daily Dry Matter Intake from the 4-fold Cross-Validation Strategy. Table S12. Dispersion Value of Each Fold for Average Daily Gain from the 4-fold Cross-Validation Strategy. Table S13. Prediction Accuracy of Each Component of Each Fold for Average Daily Dry Matter Intake from the Leave-One-Diet-Out Cross-Validation Strategy. Table S14. Prediction Accuracy of Each Component of Each Fold for Average Daily Gain from the Leave-One-Diet-Out Cross-Validation Strategy. Table S15. Root Mean Square Error of Each Fold for Average Daily Dry Matter Intake from the Leave-One-Diet-Out Cross-Validation Strategy. Table S16. Root Mean Square Error of Each Fold for Average Daily Gain from the Leave-One-Diet-Out Cross-Validation Strategy. Table S17. Dispersion Value of Each Fold for Average Daily Dry Matter Intake from the Leave-One-Diet-Out Cross-Validation Strategy. Table S18. Dispersion Value of Each Fold for Average Daily Gain from the Leave-One-Diet-Out Cross-Validation Strategy.

## Data Availability

Phenotypic and metagenomic data can be accessed via github at the following link: https://github.com/Adamyazori/Metagenome-wide-association-study-MWAS-. Host genomic data can be obtained by request to the U.S. Meat Animal Research Center (larry.kuehn@usda.gov).
