# Supplementary figures and images for: Prediction accuracy for feed intake and body weight gain using host genomic and rumen metagenomic data in beef cattle
Source: Genet Sel Evol. 2025 Oct 30;57:64. doi: 10.1186/s12711-025-01007-8 (PMC12577351; doi:10.1186/s12711-025-01007-8)

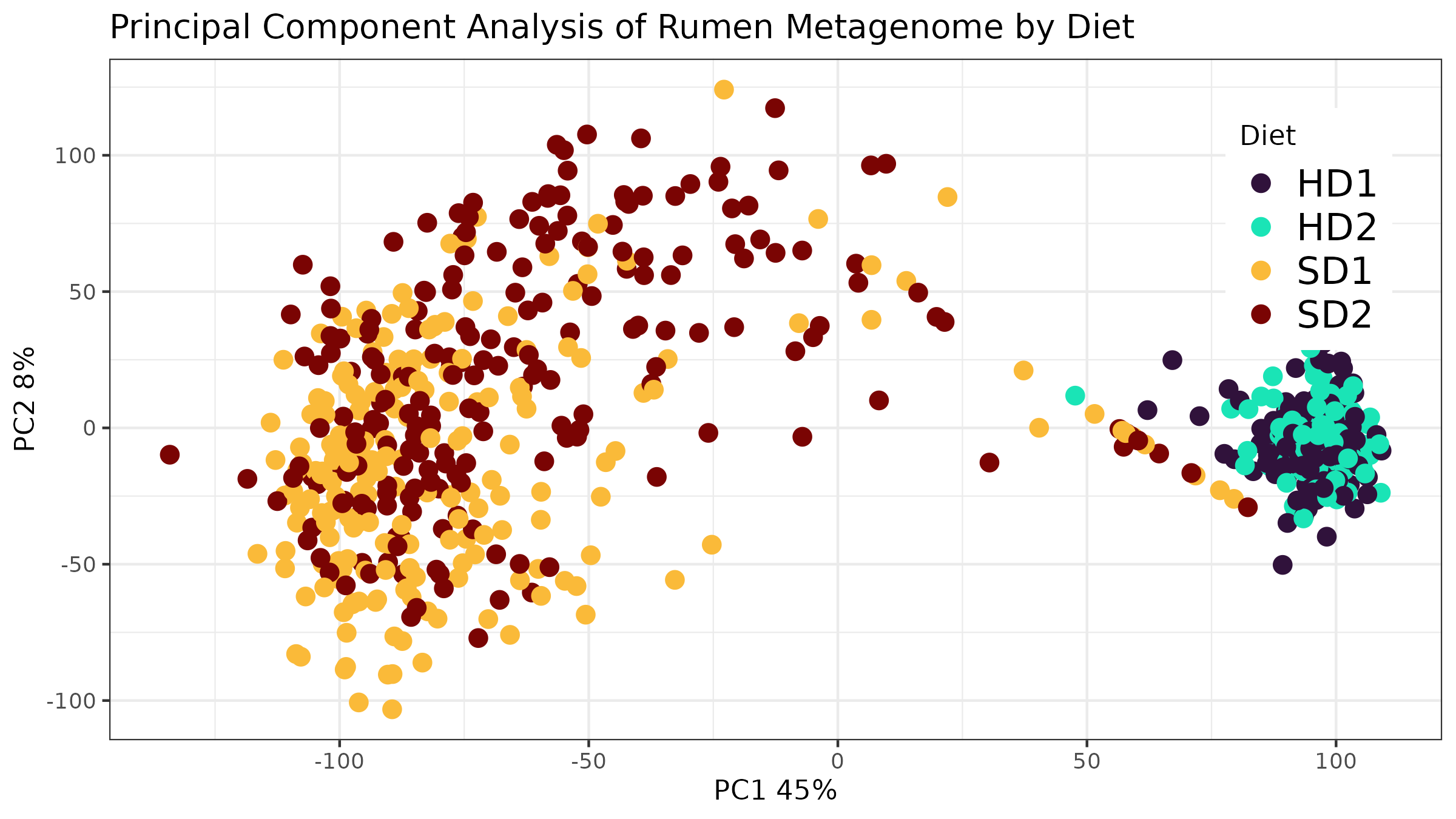

Supplement: Supplementary file 1 — Additional file 1. Figure S1. Principal Component Analysis of Rumen Metagenome by Diet. [file 12711_2025_1007_MOESM1_ESM.png]
