# Supplementary material for: Prediction accuracy for feed intake and body weight gain using host genomic and rumen metagenomic data in beef cattle
Source: Genet Sel Evol. 2025 Oct 30;57:64. doi: 10.1186/s12711-025-01007-8 (PMC12577351; doi:10.1186/s12711-025-01007-8)
Supplement: Supplementary file 2 — Additional file 2. Table S1. Variance component estimates for average daily dry matter intake (ADDMI) using host genomic and rumen metagenomic data. Table S2. Variance component estimates for average daily gain (ADG) using host genomic and rumen metagenomic data. Table S3. Median prediction accuracy (standard deviation) of 4-fold validation for average daily dry matter intake (ADDMI). Table S4. Median prediction accuracy (standard deviation) of 4-fold validation for average daily gain (ADG). Table S5. Median prediction accuracy (standard deviation) of leave-one-out validation for average daily dry matter intake (ADDMI). Table S6. Median prediction accuracy (standard deviation) of leave-one-out validation for average daily gain (ADG). [file 12711_2025_1007_MOESM2_ESM.docx]

**Table S1 Variance component estimates for average daily dry matter intake (ADDMI) using host genomic and rumen metagenomic data**

| Method | Adjustment | Complexity | Add. Gen.^a^ | Meta^b^ | Interaction^c^ | Residual^d^ |
| --- | --- | --- | --- | --- | --- | --- |
| Genomic | Naïve | Singular | 0.524708 | - | - | 1.07253 |
| Method 1 | data-driven | Singular | - | 0.165121 | - | 1.45836 |
| Method 2 | data-driven | Singular | - | 0.123201 | - | 1.47257 |
| Method 1 | data-driven | Additive | 0.535832 | 0.184133 | - | 0.955509 |
| Method 2 | data-driven | Additive | 0.532805 | 0.142954 | - | 0.970588 |
| Method 1 | data-driven | Interaction | 0.535833 | 0.184133 | 7.34 * 10^-7^ | 0.955508 |
| Method 2 | data-driven | Interaction | 0.532806 | 0.142955 | 7.46 * 10^-7^ | 0.970586 |
| Method 1 | Naïve | Singular | - | 0.491633 | - | 1.44937 |
| Method 2 | Naïve | Singular | - | 0.278254 | - | 1.42928 |
| Method 1 | Naïve | Additive | 0.514701 | 0.519246 | - | 0.969934 |
| Method 2 | Naïve | Additive | 0.508663 | 0.286643 | - | 0.956598 |
| Method 1 | Naïve | Interaction | 0.481449 | 0.448580 | 0.174725 | 0.829478 |
| Method 2 | Naïve | Interaction | 0.500612 | 0.276286 | 0.044614 | 0.921281 |
| Method 1 | Weighted | Singular | - | 0.530198 | - | 1.44947 |
| Method 2 | Weighted | Singular | - | 0.301216 | - | 1.42930 |
| Method 1 | Weighted | Additive | 0.516425 | 0.561028 | - | 0.968296 |
| Method 2 | Weighted | Additive | 0.510925 | 0.311253 | - | 0.954204 |
| Method 1 | Weighted | Interaction | 0.482150 | 0.484405 | 0.189387 | 0.822907 |
| Method 2 | Weighted | Interaction | 0.501945 | 0.299192 | 0.053719 | 0.914274 |

^a^Additive genetic variance

^b^Metagenomic variance

^c^Variance attritubed to genomic and metagenomic interaction term

^d^Residual variance

**Table S2 Variance component estimates for average daily gain (ADG) using host genomic and rumen metagenomic data**

| Method | Adjustment | Complexity | Add. Gen.^a^ | Meta^b^ | Interaction^c^ | Residual^d^ |
| --- | --- | --- | --- | --- | --- | --- |
| Genomic | Naïve | Singular | 0.0095022 | - | - | 0.0425925 |
| Method 1 | data-driven | Singular | - | 2.23 * 10^-8^ | - | 0.0513418 |
| Method 2 | data-driven | Singular | - | 3.63 * 10^-8^ | - | 0.0513418 |
| Method 1 | data-driven | Additive | 0.0095022 | 4.91 * 10^-9^ | - | 0.0425925 |
| Method 2 | data-driven | Additive | 0.0095022 | 9.88 * 10^-9^ | - | 0.0425925 |
| Method 1 | data-driven | Interaction | 0.0102551 | 0.0061986 | 0.0148774 | 0.0243571 |
| Method 2 | data-driven | Interaction | 0.0106827 | 2.43 * 10^-9^ | 0.1626500 | 0.0264183 |
| Method 1 | Naïve | Singular | - | 0.0435467 | - | 0.0450652 |
| Method 2 | Naïve | Singular | - | 0.0256239 | - | 0.0427390 |
| Method 1 | Naïve | Additive | 0.0098049 | 0.0450303 | - | 0.0358744 |
| Method 2 | Naïve | Additive | 0.0103123 | 0.0260402 | - | 0.0331333 |
| Method 1 | Naïve | Interaction | 0.0096728 | 0.0448692 | 0.0058649 | 0.0299679 |
| Method 2 | Naïve | Interaction | 0.0092703 | 0.0195249 | 0.0239955 | 0.0118923 |
| Method 1 | weighted | Singular | - | 0.0451835 | - | 0.0452796 |
| Method 2 | weighted | Singular | - | 0.0271062 | - | 0.0429211 |
| Method 1 | weighted | Additive | 0.0099461 | 0.0471937 | - | 0.0359163 |
| Method 2 | weighted | Additive | 0.0104842 | 0.0277478 | - | 0.0331087 |
| Method 1 | weighted | Interaction | 0.0098210 | 0.0473616 | 0.0059270 | 0.0301881 |
| Method 2 | weighted | Interaction | 0.0094604 | 0.0214433 | 0.0256685 | 0.0117067 |

^a^Additive genetic variance

^b^Metagenomic variance

^c^Variance attritubed to genomic and metagenomic interaction term

^d^Residual variance

**Table S3 Median prediction accuracy (standard deviation) of 4-fold validation with 5 replicates for average daily dry matter intake (ADDMI)**

| Method | Adjustment | Complexity | TAM^a^ | EBV^b^ | EMV^c^ | EIV^d^ |
| --- | --- | --- | --- | --- | --- | --- |
| Genomic | naïve | Singular | 0.16 (0.07) | 0.16 (0.07) |  |  |
| Method 1 | data-driven | Singular | 0.14 (0.05) |  | 0.14 (0.05) |  |
| Method 2 | data-driven | Singular | 0.14 (0.06) |  | 0.14 (0.06) |  |
| Method 1 | data-driven | Additive | 0.20 (0.05) | 0.17 (0.07) | 0.15 (0.05) |  |
| Method 2 | data-driven | Additive | 0.20 (0.05) | 0.17 (0.07) | 0.15 (0.06) |  |
| Method 1 | data-driven | Interaction | 0.20 (0.05) | 0.17 (0.07) | 0.15 (0.05) | 0.03 (0.05) |
| Method 2 | data-driven | Interaction | 0.20 (0.05) | 0.17 (0.07) | 0.15 (0.06) | 0.02 (0.05) |
| Method 1 | naïve | Singular | 0.14 (0.05) |  | 0.14 (0.05) |  |
| Method 2 | naïve | Singular | 0.15 (0.06) |  | 0.15 (0.06) |  |
| Method 1 | naïve | Additive | 0.28 (0.06) | 0.16 (0.07) | 0.24 (0.07) |  |
| Method 2 | naïve | Additive | 0.26 (0.06) | 0.16 (0.07) | 0.22 (0.06) |  |
| Method 1 | naïve | Interaction | 0.26 (0.06) | 0.16 (0.07) | 0.22 (0.06) | 0.11 (0.05) |
| Method 2 | naïve | Interaction | 0.26 (0.06) | 0.16 (0.07) | 0.22 (0.06) | 0.10 (0.05) |
| Method 1 | weighted | Singular | 0.26 (0.05) |  | 0.26 (0.05) |  |
| Method 2 | weighted | Singular | 0.25 (0.05) |  | 0.25 (0.05) |  |
| Method 1 | weighted | Additive | 0.28 (0.06) | 0.16 (0.07) | 0.24 (0.07) |  |
| Method 2 | weighted | Additive | 0.26 (0.06) | 0.16 (0.07) | 0.22 (0.06) |  |
| Method 1 | weighted | Interaction | 0.28 (0.05) | 0.15 (0.09) | 0.23 (0.01) | 0.13 (0.07) |
| Method 2 | weighted | Interaction | 0.26 (0.06) | 0.16 (0.07) | 0.22 (0.06) | 0.11 (0.05) |

^a^Total animal merit, the sum of solutions for all random effects included in the model

^b^Estimated breeding value, the solution for random host genetic effects

^c^Estimated microbiome value, the solution for random microbiome effects

^d^Estimated interaction value, the solution for random interaction effects

**Table S4 Median prediction accuracy (standard deviation) of 4-fold validation with 5 replicates for average daily gain (ADG)**

| Method | Adjustment | Complexity | TAM^a^ | EBV^b^ | EMV^c^ | EIV^d^ |
| --- | --- | --- | --- | --- | --- | --- |
| Genomic | naïve | Singular | 0.05 (0.08) | 0.05 (0.08) |  |  |
| Method 1 | data-driven | Singular | -0.01 (0.06) |  | -0.01 (0.06) |  |
| Method 2 | data-driven | Singular | 0.00 (0.07) |  | 0.00 (0.07) |  |
| Method 1 | data-driven | Additive | 0.03 (0.08) | 0.06 (0.08) | -0.01 (0.06) |  |
| Method 2 | data-driven | Additive | 0.01 (0.08) | 0.06 (0.08) | -0.01 (0.07) |  |
| Method 1 | data-driven | Interaction | 0.06 (0.07) | 0.06 (0.08) | 0.00 (0.07) | -0.01 (0.06) |
| Method 2 | data-driven | Interaction | 0.01 (0.08) | 0.07 (0.08) | 0.01 (0.08) | 0.00 (0.06) |
| Method 1 | naïve | Singular | 0.08 (0.06) |  | 0.08 (0.06) |  |
| Method 2 | naïve | Singular | 0.11 (0.06) |  | 0.11 (0.06) |  |
| Method 1 | naïve | Additive | 0.08 (0.06) | 0.04 (0.08) | 0.08 (0.06) |  |
| Method 2 | naïve | Additive | 0.11 (0.06) | 0.04 (0.08) | 0.11 (0.06) |  |
| Method 1 | naïve | Interaction | 0.08 (0.06) | 0.08 (0.06) | 0.07 (0.06) | 0.11 (0.05) |
| Method 2 | naïve | Interaction | 0.11 (0.06) | 0.04 (0.08) | 0.08 (0.06) | 0.11 (0.06) |
| Method 1 | weighted | Singular | 0.08 (0.07) |  | 0.08 (0.07) |  |
| Method 2 | weighted | Singular | 0.11 (0.06) |  | 0.11 (0.06) |  |
| Method 1 | weighted | Additive | 0.08 (0.06) | 0.04 (0.08) | 0.08 (0.06) |  |
| Method 2 | weighted | Additive | 0.11 (0.05) | 0.04 (0.08) | 0.11 (0.06) |  |
| Method 1 | weighted | Interaction | 0.08 (0.06) | 0.04 (0.08) | 0.07 (0.06) | 0.11 (0.05) |
| Method 2 | weighted | Interaction | 0.11 (0.06) | 0.08 (0.08) | 0.08 (0.06) | 0.09 (0.06) |

^a^Total animal merit, the sum of solutions for all random effects included in the model

^b^Estimated breeding value, the solution for random host genetic effects

^c^Estimated microbiome value, the solution for random microbiome effects

^d^Estimated interaction value, the solution for random interaction effects

**Table S5 Median prediction accuracy (standard deviation) of leave-one-diet-out validation for average daily dry matter intake (ADDMI)**

| Method | Adjustment | Complexity | TAM^a^ | EBV^b^ | EMV^c^ | EIV^d^ |
| --- | --- | --- | --- | --- | --- | --- |
| Genomic | naïve | Singular | 0.08 (0.06) | 0.08 (0.06) |  |  |
| Method 1 | data-driven | Singular | 0.08 (0.14) |  | 0.08 (0.14) |  |
| Method 2 | data-driven | Singular | 0.12 (0.11) |  | 0.12 (0.011) |  |
| Method 1 | data-driven | Additive | 0.09 (0.08) | 0.10 (0.06) | 0.09 (0.13) |  |
| Method 2 | data-driven | Additive | 0.10 (0.09) | 0.10 (0.06) | 0.13 (0.10) |  |
| Method 1 | data-driven | Interaction | 0.09 (0.08) | 0.10 (0.06) | 0.09 (0.13) | -0.01 (0.02) |
| Method 2 | data-driven | Interaction | 0.10 (0.09) | 0.10 (0.06) | 0.13 (0.10) | -0.01 (0.05) |
| Method 1 | naïve | Singular | 0.14 (0.11) |  | 0.14 (0.11) |  |
| Method 2 | naïve | Singular | 0.16 (0.14) |  | 0.16 (0.14) |  |
| Method 1 | naïve | Additive | 0.11 (0.09) | 0.10 (0.06) | 0.15 (0.11) |  |
| Method 2 | naïve | Additive | 0.14 (0.09) | 0.10 (0.06) | 0.16 (0.14) |  |
| Method 1 | naïve | Interaction | 0.14 (0.08) | 0.10 (0.06) | 0.12 (0.09) | 0.07 (0.04) |
| Method 2 | naïve | Interaction | 0.13 (0.09) | 0.10 (0.06) | 0.16 (0.14) | 0.10 (0.03) |
| Method 1 | weighted | Singular | 0.13 (0.11) |  | 0.13 (0.11) |  |
| Method 2 | weighted | Singular | 0.16 (0.14) |  | 0.16 (0.14) |  |
| Method 1 | weighted | Additive | 0.12 (0.09) | 0.10 (0.06) | 0.15 (0.11) |  |
| Method 2 | weighted | Additive | 0.14 (0.09) | 0.10 (0.06) | 0.16 (0.14) |  |
| Method 1 | weighted | Interaction | 0.14 (0.08) | 0.10 (0.06) | 0.11 (0.09) | 0.07 (0.04) |
| Method 2 | weighted | Interaction | 0.14 (0.09) | 0.10 (0.06) | 0.16 (0.14) | 0.08 (0.03) |

^a^Total animal merit, the sum of solutions for all random effects included in the model

^b^Estimated breeding value, the solution for random host genetic effects

^c^Estimated microbiome value, the solution for random microbiome effects

^d^Estimated interaction value, the solution for random interaction effects

**Table S6 Median prediction accuracy (standard deviation) of leave-one-diet-out validation for average daily gain (ADG)**

| Method | Adjustment | Complexity | TAM^a^ | EBV^b^ | EMV^c^ | EIV^d^ |
| --- | --- | --- | --- | --- | --- | --- |
| Genomic | naïve | Singular | 0.04 (0.10) | 0.04 (0.10) |  |  |
| Method 1 | data-driven | Singular | 0.05 (0.06) |  | 0.05 (0.06) |  |
| Method 2 | data-driven | Singular | 0.03 (0.11) |  | 0.03 (0.11) |  |
| Method 1 | data-driven | Additive | 0.03 (0.09) | 0.03 (0.10) | 0.04 (0.07) |  |
| Method 2 | data-driven | Additive | 0.01 (0.11) | 0.03 (0.10) | 0.04 (0.11) |  |
| Method 1 | data-driven | Interaction | 0.06 (0.11) | 0.03 (0.10) | 0.06 (0.07) | 0.02 (0.11) |
| Method 2 | data-driven | Interaction | 0.04 (0.12) | 0.05 (0.10) | 0.06 (0.10) | -0.03 (0.11) |
| Method 1 | naïve | Singular | 0.06 (0.06) |  | 0.06 (0.06) |  |
| Method 2 | naïve | Singular | 0.10 (0.07) |  | 0.10 (0.07) |  |
| Method 1 | naïve | Additive | 0.08 (0.03) | 0.04 (0.09) | 0.06 (0.05) |  |
| Method 2 | naïve | Additive | 0.08 (0.08) | 0.03 (0.09) | 0.10 (0.07) |  |
| Method 1 | naïve | Interaction | 0.09 (0.06) | 0.04 (0.09) | 0.06 (0.05) | 0.12 (0.08) |
| Method 2 | naïve | Interaction | 0.15 (0.10) | 0.03 (0.09) | 0.08 (0.07) | 0.11 (0.09) |
| Method 1 | weighted | Singular | 0.06 (0.06) |  | 0.06 (0.06) |  |
| Method 2 | weighted | Singular | 0.10 (0.07) |  | 0.10 (0.07) |  |
| Method 1 | weighted | Additive | 0.08 (0.03) | 0.04 (0.09) | 0.06 (0.05) |  |
| Method 2 | weighted | Additive | 0.08 (0.08) | 0.03 (0.09) | 0.10 (0.07) |  |
| Method 1 | weighted | Interaction | 0.08 (0.06) | 0.04 (0.09) | 0.06 (0.05) | 0.12 (0.08) |
| Method 2 | weighted | Interaction | 0.15 (0.10) | 0.03 (0.09) | 0.08 (0.07) | 0.11 (0.09) |

^a^Total animal merit, the sum of solutions for all random effects included in the model

^b^Estimated breeding value, the solution for random host genetic effects

^c^Estimated microbiome value, the solution for random microbiome effects

^d^Estimated interaction value, the solution for random interaction effects
